# Statistical Issues and Lessons Learned From COVID-19 Clinical Trials With Lopinavir-Ritonavir and Remdesivir

**DOI:** 10.2196/19538

**Published:** 2020-07-10

**Authors:** Guosheng Yin, Chenyang Zhang, Huaqing Jin

**Affiliations:** 1 Department of Statistics and Actuarial Science The University of Hong Kong Hong Kong China (Hong Kong)

**Keywords:** coronavirus, COVID-19, cure rate model, sample size adjustment, terminal event, type I error rate, restricted mean survival time

## Abstract

**Background:**

Recently, three randomized clinical trials on coronavirus disease (COVID-19) treatments were completed: one for lopinavir-ritonavir and two for remdesivir. One trial reported that remdesivir was superior to placebo in shortening the time to recovery, while the other two showed no benefit of the treatment under investigation.

**Objective:**

The aim of this paper is to, from a statistical perspective, identify several key issues in the design and analysis of three COVID-19 trials and reanalyze the data from the cumulative incidence curves in the three trials using more appropriate statistical methods.

**Methods:**

The lopinavir-ritonavir trial enrolled 39 additional patients due to insignificant results after the sample size reached the planned number, which led to inflation of the type I error rate. The remdesivir trial of Wang et al failed to reach the planned sample size due to a lack of eligible patients, and the bootstrap method was used to predict the quantity of clinical interest conditionally and unconditionally if the trial had continued to reach the originally planned sample size. Moreover, we used a terminal (or cure) rate model and a model-free metric known as the restricted mean survival time or the restricted mean time to improvement (RMTI) to analyze the reconstructed data. The remdesivir trial of Beigel et al reported the median recovery time of the remdesivir and placebo groups, and the rate ratio for recovery, while both quantities depend on a particular time point representing local information. We use the restricted mean time to recovery (RMTR) as a global and robust measure for efficacy.

**Results:**

For the lopinavir-ritonavir trial, with the increase of sample size from 160 to 199, the type I error rate was inflated from 0.05 to 0.071. The difference of RMTIs between the two groups evaluated at day 28 was –1.67 days (95% CI –3.62 to 0.28; *P*=.09) in favor of lopinavir-ritonavir but not statistically significant. For the remdesivir trial of Wang et al, the difference of RMTIs at day 28 was –0.89 days (95% CI –2.84 to 1.06; *P*=.37). The planned sample size was 453, yet only 236 patients were enrolled. The conditional prediction shows that the hazard ratio estimates would reach statistical significance if the target sample size had been maintained. For the remdesivir trial of Beigel et al, the difference of RMTRs between the remdesivir and placebo groups at day 30 was –2.7 days (95% CI –4.0 to –1.2; *P*<.001), confirming the superiority of remdesivir. The difference in the recovery time at the 25th percentile (95% CI –3 to 0; *P*=.65) was insignificant, while the differences became more statistically significant at larger percentiles.

**Conclusions:**

Based on the statistical issues and lessons learned from the recent three clinical trials on COVID-19 treatments, we suggest more appropriate approaches for the design and analysis of ongoing and future COVID-19 trials.

## Introduction

### Background

The novel coronavirus disease (COVID-19) has spread all over the world at an unprecedented rate since its outbreak in December 2019. More than 200 countries or territories have confirmed cases, and over 8.4 million individuals have been infected, leading to more than 45,0000 deaths as of June 18, 2020. COVID-19 was declared a Public Health Emergency of International Concern by the World Health Organization (WHO) on January 30 and declared a pandemic on March 11, 2020.

As recommended by the WHO R&D Blueprint expert group, clinical improvements for patients with COVID-19 can be classified in a seven-category ordinal scale [1]:

1. Not hospitalized with resumption of normal activities
2. Not hospitalized, but unable to resume normal activities
3. Hospitalized, not requiring supplemental oxygen
4. Hospitalized, requiring supplemental oxygen
5. Hospitalized, requiring nasal high-flow oxygen therapy, noninvasive mechanical ventilation, or both
6. Hospitalized, requiring extracorporeal membrane oxygenation, invasive mechanical ventilation, or both
7. Death

So far, there are only eight clinical trials for COVID-19 completed with results published. Among them, two trials were for hydroxychloroquine with relatively small sample sizes (30 patients for the trial of Chen et al [2] and 36 patients for the trial of Gautret et al [3]). Although the trial conducted by Gautret et al [3] yielded a significant result, the sample size was too small to draw any convincing conclusion. The trial of Cai et al [4] compared favipiravir and lopinavir-ritonavir with a total sample size of 80 patients, leading to a significant result (*P*=.004). Chen et al [5] conducted a trial comparing favipiravir with arbidol, which had a total sample size of 240 patients and yielded an insignificant result. The trial of Grein et al [6] was a single-arm trial for remdesivir, and the estimated clinical improvement rate at day 18 was 0.68. To determine the efficacy of Lianhuaqingwen (LHQW) capsule, a compounded Chinese herb medicine, Hu et al [7] conducted an open-label randomized controlled trial and reported a statistically significant difference in the symptom (fever, fatigue, coughing) recovery rate between the treatment group and the control group (91.5% vs 82.4%; *P*=.022). However, the trial did not include a placebo in the control group to implement a double-blinding scheme. Despite the urgency nature of the pandemic, their argument for unblinding due to ethical reasons seems to be unsound. Due to the conscious and subconscious psychological tendencies of humans including both clinicians and patients, bias often arises in an open-label study. Not only does unblinding lead to potential selection bias, but it may also cause placebo effects for patients who took LHQW [8-11], which thus shed doubts on the clinical benefits of LHQW. In particular, the rate of symptom recovery is related to disease relief or symptomatic manifestations such as fever, fatigue, and coughing (“soft” end points), for which placebo effects are known to be strong and more discernible [10]. However, the LHQW and control groups did not differ in the rate of conversion to severe cases or viral assay findings (“hard” end points), for which placebo effects are less perceptible because generally placebos can neither alter the pathophysiology of the disease nor cure it. We take the three randomized clinical trials conducted by Cao et al [12] on lopinavir-ritonavir and by Wang et al [13] and Beigel et al [14] on remdesivir as examples to illustrate statistical issues and lessons learned, as they have drawn great attention in the clinical community.

### Lopinavir-Ritonavir Trial

The Lopinavir Trial for Suppression of Severe Acute Respiratory Syndrome Coronavirus 2 in China [12] was conducted with record speed from January 18 to February 3, 2020 (the date of enrollment of the last patient). Patient recruitment up to a planned sample size is often the bottle neck of trial conduct. This was not the case with severe COVID-19 due to the abundance of hospitalized patients during that period of time. In this trial, eligible patients were randomized at a 1:1 ratio to either the lopinavir-ritonavir treatment group (400 mg and 100 mg orally, twice daily) plus the standard care or the standard care alone for 14 days. No placebo was used for blinding because no placebo was prepared due to the urgency of the trial; therefore, both patients and investigators were aware of the treatment identity each patient received. Following the WHO seven-ordinal scale [1], the primary end point adopted by the trial [12] was the time to clinical improvement, which was defined as the time from randomization to an improvement of two points from the status at randomization (eg, from point 6 to point 4 or from point 5 to point 3) or live discharge from the hospital, whichever came first. The sample size was increased from 160 to 199 since the result with the enrolled 160 patients did not reach statistical significance. As a final conclusion, Cao et al [12] reported no benefit with the lopinavir-ritonavir treatment beyond the standard care with a hazard ratio (HR) of 1.24 and the associated 95% CI 0.90-1.72.

### Remdesivir Trial 1

Wang et al [13] conducted a randomized, double-blind, placebo-controlled, multicenter trial with remdesivir at ten hospitals in Hubei, China. Overall, 236 patients were enrolled from February 6 to March 12, 2020, and were randomly assigned to the remdesivir group (200 mg on day 1 followed by 100 mg on days 2-10) and the placebo group at a 2:1 ratio. In the original design, the trial planned to recruit 453 patients with 302 to remdesivir and 151 to placebo, but no patients were enrolled after March 12 due to no eligible patients being available in the Hubei Province. As a consequence, the statistical power of the study was reduced from 80% to 58%. The primary clinical end point was the time to improvement within 28 days. Clinical improvement was defined as a two-point improvement from an adjusted six-category ordinal scale from the WHO seven-category ordinal scale. In conclusion, remdesivir did not show statistically significant clinical benefit compared with the placebo in terms of the HR 1.23 (95% CI 0.87-1.75).

### Remdesivir Trial 2

Beigel et al [14] reported a randomized, double-blind, placebo-controlled trial of intravenous remdesivir in adults hospitalized with COVID-19 and evidence of lower respiratory tract infection. This trial had a total sample size of 1059 patients (538 assigned to remdesivir and 521 to placebo). The median recovery time of the remdesivir group was 11 (95% CI 9-12) days and 15 (95% CI 13-19) days for the placebo group. The rate ratio for recovery was 1.32 (95% CI 0.47-1.04; *P*<.001), which was statistically significant in favor of remdesivir. The Kaplan-Meier estimates of mortality at 14 days were 7.1% with remdesivir and 11.9% with the placebo, and the HR for death was 0.70 (95% CI 0.47-1.04). Remdesivir was shown to be superior to the placebo in shortening the time to recovery in adults hospitalized with COVID-19, and, in terms of the HR for death, there was no significant difference between the two groups.

So far, only one treatment, remdesivir, has been shown to be effective by a randomized clinical trial, but the other remdesivir trial failed to demonstrate its superiority over the placebo. As the pandemic of COVID-19 will not be controlled anytime soon, the aforementioned three clinical trials [12-14] provide extremely valuable information on the treatments of COVID-19 and the corresponding trial design and analysis. However, several important issues have been identified in the statistical analysis, design, and implementation of the three trials. We point out the statistical problems that arose in the three trials [12-14] and reanalyze the data from the cumulative incidence curves for the time to improvement or recovery using more appropriate approaches. Our in-depth and comprehensive analyses yield new insights on the design and analysis for ongoing and future COVID-19 clinical trials.

## Methods

### Inflation of the Type I Error

The log-rank test [15] is the most commonly used method in survival analysis and clinical trial design to compare the survival benefit of two arms. Consider a randomized clinical trial with a planned sample size *N*_1_ using a two-sided log-rank test. If the hypothesis test indicates no significant survival difference between the two groups under the significance level *α* but the trial decides to continue to enroll more patients up to a larger sample size *N*_2_, this would inflate the overall type I error of the trial. Any adjustment to the sample size during the trial should be planned and evaluated in advance to maintain the overall type I error rate.

Let *Z*_1_ and *Z*_2_ denote the log-rank test statistics with sample sizes *N*_1_ and *N*_2_, respectively. It holds that under the null hypothesis [16,17] *Z*_1_ and *Z*_2_ jointly follow a multivariate normal distribution:


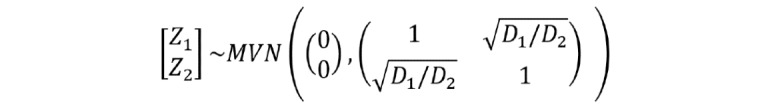
 (**1**)

*D*_1_ = *dN*_1_ and *D*_2_ = *dN*_2_ are the expected numbers of events with sample sizes *N*_1_ and *N*_2_, and *d* is the proportion of patients experiencing the event. Thus, the overall type I error rate *α* overall with the significance level *α* is:


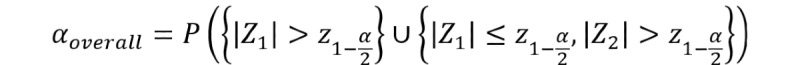

(**2**)


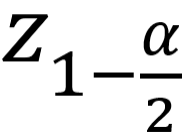
 is the (1 – 
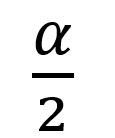
)th quantile of the standard normal distribution.

### Terminal (or Cure) Rate Model

For clinical studies with a survival end point, we are interested in the distribution of event time *T*. In general, patients will eventually experience the event with a long enough follow-up; although, the exact event time might not be observed due to censoring. However, for some diseases with long-term survivors, it may happen that the event will never occur in a fraction of subjects (ie, the event time for cured subjects is infinity [18-21]). Under this situation, patients can be divided into two groups: the terminal (or cure) group (the specified event would never occur) and the nonterminal group (the specified event would occur but possibly censored due to the end time of the study). Thus, the distribution of the event time *T* has a point probability mass η at ∞:

*T* = (1 –η)T^*^ + η∞ (**3**)

*η* is the group label taking a value of 1 if the individual is in the terminal group and 0 otherwise; γ = *P*(η = 1) = *P*(*T* = ∞) is the terminal rate and T^*^ follows a proper distribution with *P*(T^*^ < ∞) = 1. For the COVID-19 trials [12,13], the cumulative incidence curve of *T* can be expressed by


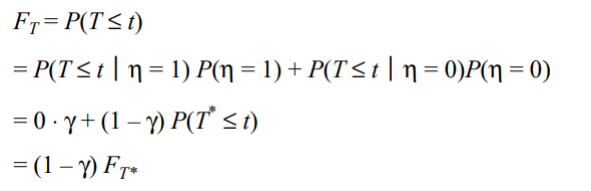
 (**4**)

*F_T_* and F_T__*_ are the cumulative distribution functions of *T* and T*, respectively. Note that *P*(*T* < ∞) = 1 – γ < 1.

### Restricted Mean Survival Time

Restricted mean survival time (RMST) [16,22-26] is an alternative measure for the mean survival time that is not estimable due to the presence of censoring. The RMST is equal to the expectation of the minimum value of event time *T* and the specified time point *τ*, which can be calculated as the area under the survival curve from 0 to *τ*. It can be estimated by the area under the Kaplan-Meier survival curve, which has gained enormous popularity due to its robustness feature.

Although the HR is the most popular statistic to quantify the survival difference in randomized clinical trials, it is no longer an interpretable quantity if the proportional hazards (PH) assumption is violated [25]. By contrast, the RMST has the advantages of being nonparametric and model-free yet carrying clinically meaningful interpretations. Given the prespecified time point *τ*, the estimate of the RMST difference between two groups can be interpreted as the extra survival gain on average during the time *τ* follow-up period.

### Predicted Trial Outcome With Sample Size Projection

Clinical trials during the epidemic of an infectious disease might fail to reach the planned sample size due to a lack of eligible patients if the outbreak can be quickly controlled [27]. However, early termination of a clinical trial would inevitably lead to loss of power and thus unconvincing findings. Based on the collected data, the bootstrap method can be used to predict what would happen if the trial had continued to reach the desired sample size. Let *N* denote the desired sample size and *N*_0_ (*N*_0_ < *N*) the actual number of patients enrolled. The statistic of interest prediction can be conducted under either conditional or unconditional schemes. The unconditional prediction draws *N* samples (sampling with replacement from the original data with *N*_0_ observations), while the conditional prediction draws *N* – *N*_0_ samples from the original *N*_0_ observations and keeps the original *N*_0_ samples intact. By repeating the sampling procedure for a large number of times, one can estimate the predicted mean and the corresponding confidence interval for the statistic of interest if the trial had continued to reach the sample size of *N*.

## Results

### Lopinavir-Ritonavir Trial of Cao et al

In the original analysis of Cao et al [12], the time to clinical improvement was assessed after all patients had reached day 28, and failure to reach clinical improvement or death before day 28 were considered as right-censored at day 28. In contrast to the usual survival analysis where death (or a bad event such as disease progression) is used as the event of interest, a good event (clinical improvement) was adopted as the end point in this trial. As a result, the shorter time to reach clinical improvement, the better. Cao et al [12] concluded no benefit of using the lopinavir-ritonavir treatment beyond the standard care with an HR of 1.24 (95% CI 0.90-1.72).

We carried out an in-depth and comprehensive investigation of the trial design in Cao et al [12] and identified several key issues with the trial that might have hindered its success. First, the unplanned sample size increment from 160 to 199 would inflate the type I error rate. For this trial, we have *N*_1_=160, *N*_2_=199, *d*=0.75, *D*_1_ = 160 × 0.75 = 120, *D*_2_ = 199 × 0.75 = 149.25, and based on equation 2, *α*_overall_=.071 when the nominal significance level is set as *α*=.05. That is, the false-positive rate for this trial increased as high as 7.1% in contrast to the nominal level of 5%. Any sample size alteration or re-estimation should be planned in advance to control the type I error rate and maintain the integrity of a trial. When the sample size reached 199, the trial was halted for enrollment because of the availability of another treatment, remdesivir. Such termination of a trial was again unplanned and immature; if there were not another agent available, would the trial continue recruitment? Interestingly, the remdesivir trial by Wang et al [13] (the same group of investigators as the lopinavir-ritonavir trial) started 3 days later after the lopinavir-ritonavir trial was terminated.

In terms of the primary end point, clinical improvement using two-level increment on a seven-category ordinal scale from baseline is ad hoc due to uneven clinical differences between adjacent scales. For example, it is ambiguous whether the status of a patient changing from point 5 to point 3 is equivalent to that of changing from point 6 to point 4. In addition, live discharge from the hospital may occur from point 3 to point 2 or point 4 to point 2, which cannot be considered equivalent either. Thus, choosing 2-point improvement on the clinical outcome scale is not a precise end point, which ignores the 1-point improvement and the difference between 2-point and 3-point improvement. Instead, we recommend death as a single and clean end point for such trials, given the mortality rate was not low with patients who were hospitalized with severe COVID-19 (19.2% in the lopinavir-ritonavir group and 25.0% in the standard care group).

The original analysis [12] treated death before day 28 as right-censored at day 28, no matter when death had occurred. This may cause ambiguity because it cannot distinguish the situations where all deaths in one group occurred earlier while those in the other group occurred later. As death is a terminal event, a terminal (or cure) rate model would be more appropriate for analysis of such data. A terminal rate model can be viewed as the counterpart of the traditional mixture cure rate model [18-21], which can be developed by slight modifications. As death is a terminal event, patients who died during the 28-day follow-up period would never reach the clinical improvement (ie, the time to clinical improvement was infinity) denoted as ∞. Death can also be viewed as a competing risk for clinical improvement.

The upper panel of Table 1 shows that there was neither any significant difference in the terminal rates between the lopinavir-ritonavir and standard care groups or in the HR (after excluding the terminal subjects who would eventually be absorbed in the death state) from the mixture terminal rate model. In particular, the terminal rates (including observed deaths as well as unobserved deaths that would occur after day 28 but were censored at day 28) were 21.17% for the lopinavir-ritonavir group and 29.91% for the standard care group with *P*=.16, and the HR for nonterminal subjects was 1.05 (95% CI 0.78-1.42; *P*=.74).

**Figure 1:**
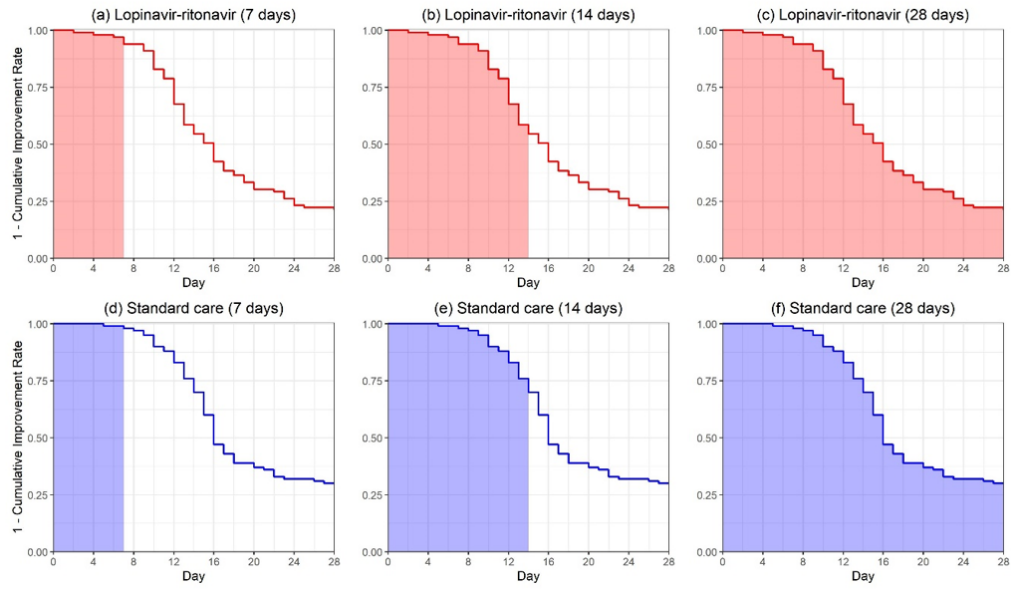
The restricted mean time to improvement corresponding to the area under the curves for the lopinavir-ritonavir group and the standard care group evaluated at days 7, 14, and 28 in Cao et al [12].

Moreover, the crossings of the cumulative event curves for the lopinavir-ritonavir and standard care groups at days 10 and 16 in the second figure of Cao et al [12] imply possible violation of the PH assumption. When the PH assumption is not satisfied, the HR from a Cox model [29] is not clinically meaningful. As an alternative, the area above the curve in the second figure of Cao et al [12] or the area under the inverted curve as shown in our Figure 1, referred to as the restricted mean time to improvement (RMTI), can be used to quantify treatment effect that requires no assumption such as PH [16,22-26]. As a model-free quantity, the RMTI up to 28 days can be interpreted as the average time to reach improvement in 28 days, for which the shorter is the better. The 28-day RMTI difference between the two groups was 1.67 days (95% CI –3.62 to 0.28; *P*=.09) in favor of lopinavir-ritonavir but not statistically significant. The 7-day and 14-day RMTIs are also presented in the lower panel of Table 1, where the 14-day RMTI showed some promising results for lopinavir-ritonavir, yet further confirmation is needed.

Tables 2 and 3 show the numbers on mortality and clinical improvement by day 28 across the two treatment groups, respectively. We carried out chi-square tests (or Fisher exact tests if some of the cell counts were smaller than 5) to examine any association between the outcomes and treatments. For Table 2 with 2×3 cells, there is no association with *P*=.53, and if combining deaths in both earlier and later stages, this leads to 2×2 cells with *P*=.32 and odds ratio 0.71 (95% CI 0.36-1.40). Patients treated with lopinavir-ritonavir had 0.71 times odds to die by day 28 in comparison to those in the standard care group. For Table 3 with 2×4 cells, there is no association with *P*=.11, and if combining all clinical improvement cases, this leads to 2×2 cells with *P*=.53 and odds ratio 1.24 (95% CI 0.64-2.40). Patients treated with lopinavir-ritonavir had 1.24 times odds to achieve clinical improvement by day 28 in comparison to those in the standard care group. However, none of the results are statistically significant.

**Table 1:** Comparisons of estimates from the mixture terminal (or cure) model and the RMTI based on the reconstructed data from the second figure in Cao et al [12].^a^

Terminal rate model^b^		Lopinavir-ritonavir	Standard care	Difference	*P* value	Hazard ratio (95% CI)	*P* value
Terminal rate, % (95% CI)		21.17 (15.77-28.42)	29.91 (4.40-36.66)	–8.74 (–21.04 to 3.55)	.16	1.05 (0.78-1.42)	.74
**RMTI^c^ (95% CI)**							
	Day 7	6.91 (6.79-7.00)	6.98 (6.94-7.00)	–0.07 (–0.19 to 0.05)	.26	N/A^d^	N/A
	Day 14	12.58 (12.11-13.04)	13.25 (12.92-13.58)	–0.67 (–1.24 to –0.11)	.02	N/A	N/A
	Day 28	17.19 (15.78-18.60)	18.86 (17.51-20.21)	–1.67 (3.62 to 0.28)	.09	N/A	N/A

^a^Cumulative incidence curves were extracted and reconstructed from the second figure in Cao et al [12] using the “digitize” package [28] in R software (R Foundation for Statistical Computing).

^b^The mixture terminal rate model was performed using the “smcure” package.

^c^The RMTI (restricted mean time to improvement) was estimated by calculating the area above the cumulative incidence curve using the “survRM2” package.

^d^Not applicable.

**Table 2:** Counts of deaths for the earlier stage (≤12 days after onset of symptoms) and later stage (>12 days after onset of symptoms), and survivors.

Treatment	Deaths		Survivors, n	
	Earlier, n	Later, n		
Lopinavir-ritonavir	8	11		80
Standard care	13	12		75

**Table 3:** Counts of clinical improvement cases in days 1-7, 8-14, and 15-28, and nonimprovement cases.

Treatment	Clinical Improvement				No improvement, n
	Days 1-7, n	Days 8-14, n	Days 15-28, n		
Lopinavir-ritonavir	6	39	33	22	
Standard care	2	28	40	30	

### Remdesivir Trial of Wang et al

Wang et al [13] reported a randomized, double-blind, placebo-controlled remdesivir trial for patients with severe COVID-19. Based on an adjusted six-point ordinal scale of clinical status, the primary end point was the time to clinical improvement, defined as a 2-level decline from randomization (similar to that in Cao et al [12]; in fact, the two trials were conducted by the same group of investigators), for which the shorter is the better. Patients were permitted concomitant use of lopinavir-ritonavir, interferons, and corticosteroids. The HR between the remdesivir and placebo groups was 1.23 (95% CI 0.87-1.75), indicating no significant difference. Overall, 237 eligible patients were enrolled, with 158 patients assigned to the remdesivir group and 78 patients to the placebo group under the intent-to-treat (ITT) scheme. The trial was stopped early and thus failed to reach the designated sample size 453 due to a lack of eligible patients.

Similar to the trial by Cao et al [12], deaths before day 28 were treated as right-censored observations at day 28, regardless of the actual occurrence time of deaths in Wang et al [13]. Moreover, a clinical improvement might not be observed due to death (ie, death is a terminal event), and thus, the terminal or cure rate model introduced earlier should be recommended for the survival analysis rather than the standard Cox model.

The upper panel of Table 4 indicates no significant difference in the terminal rates between the remdesivir and placebo groups. In particular, the terminal rates were 31.49% for the remdesivir group and 40.71% for the placebo group with *P*=.19. With the terminal subjects excluded, the HR from the mixture terminal rate model was 0.92 (95% CI 0.63-1.35; *P*=.67), which also showed no significant difference between the two groups.

**Figure 2:**
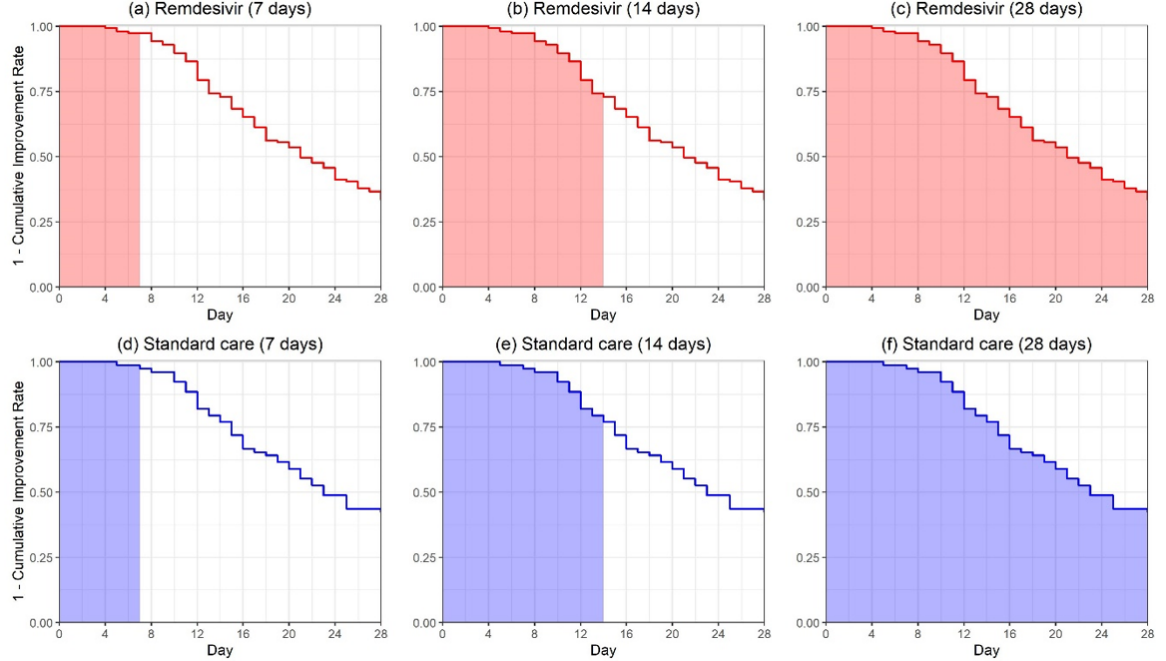
The restricted mean time to improvement corresponding to the area under the curves for the remdesivir group and the placebo group evaluated at days 7, 14, and 28 in Wang et al [13].

Due to the competing risk from death, the end point might not be observed, and thus, the standard hazard concept is ambiguous, and the HR does not have a meaningful interpretation anymore [30]. In the second figure in Wang et al [13], the curve for the cumulative improvement event of remdesivir is uniformly higher than that of the control, indicating patients with remdesivir reached improvement faster than those in the control group. The area above the cumulative incidence curve or, equivalently, the area under the survival curve up to 28 days in our Figure 2 would be a reasonable quantity for evaluating the treatment efficacy. Using the reconstructed data from the second figure in Wang et al [13], the RMTI evaluated at day 28 was 20.42 (95% CI 19.26-21.57) days for the remdesivir group and 21.31 (95% CI 19.73-22.88) days for the placebo group. As shown in the lower panel of Table 4, the difference in RMTIs was –0.89 days (95% CI –2.84 to 1.06), numerically favoring remdesivir but not statistically significant. It can be interpreted that patients treated by remdesivir on average had an extra 0.89 days of improvement during the 28-day follow-up compared with those in the placebo group. The 7-day and 14-day RMTIs are also presented in the lower panel of Table 4, and neither showed statistically significant results.

The trial was terminated without reaching the originally planned sample size, 453, due to a lack of eligible patients. With only 236 patients in the ITT analysis, the estimated HR was 1.23 (95% CI 0.87-1.75), numerically favoring remdesivir, which might not be reliable due to the underpowered study. Using the bootstrap method, we can predict what would happen if the trial had continued to reach the full sample size or double the planned sample size. Table 5 shows both the unconditional and conditional predictions of the HR, similar to sample size re-estimation using conditional power [31] in a two-stage design. If the trial could have reached the designated sample size, the HR from the conditional prediction shows the significant treatment effect of remdesivir with *P*=.02, and if the trial had enrolled twice of the target sample size, both conditional and unconditional approaches result in significant differences under the 5% significance level. Thus, a larger sample size may be needed to show the significant difference between remdesivir and placebo.

**Table 4:** Comparisons of the estimates from the mixture terminal (or cure) rate model and the RMTI based on the reconstructed data from the second figure in Wang et al [13].

Terminal rate model		Remdesivir	Placebo	Difference	*P* Value	Hazard ratio (95% CI)	*P* value
Terminal rate, % (95% CI)		0.31 (0.27-0.37)	0.41 (0.32-0.51)	–9.22 (–22.9 to 4.45)	.19	0.92 (0.63-1.35)	.67
**RMTI^a^**							
	Day 7	6.95 (6.90-7.00)	6.97 (6.92-7.00)	–0.03 (–0.10 to 0.05)	.49	N/A^b^	N/A
	Day 14	13.09 (12.78-13.40)	13.29 (12.92-13.67)	–0.20 (–0.69 to 0.29)	.42	N/A	N/A
	Day 28	20.42 (19.26-21.57)	21.31 (19.73-22.88)	–0.89 (–2.84 to 1.06)	.37	N/A	N/A

^a^RMTI: restricted mean time to improvement.

^b^Not applicable.

**Table 5:** Predicted hazard ratios (with 95% CIs) and *P* values at the actual, target, and double target sample sizes using 50,000 bootstrap samples based on the reconstructed data from the second figure in Wang et al [13].

Sample size	Sample size in each arm		Unconditional prediction		Conditional prediction	
	Remdesivir, n	Placebo, n	HR^a^ (95% CI)	*P* value	HR (95% CI)	*P* value
Actual	158	78	1.23 (0.87-1.75)	.24	N/A^b^	N/A
Target	302	151	1.24 (0.96-1.60)	.10	1.24 (1.03-1.48)	.02
Target×2	604	302	1.24 (1.03-1.48)	.02	1.24 (1.06-1.44)	.01

^a^HR: hazard ratio.

^b^Not applicable.

### Remdesivir Trial of Beigel et al

Beigel et al [14] presented a preliminary report of the NCT04280705 trial, which is a randomized, double-blind, placebo-controlled trial of intravenous remdesivir in adults hospitalized with COVID-19 and evidence of lower respiratory tract involvement. This trial enrolled 1059 patients (538 assigned to remdesivir and 521 to placebo). The primary end point of the original analysis was the recovery time, defined by either discharge from the hospital or hospitalization for infection-control purposes only. The median recovery time of the remdesivir group was 11 (95% CI 9-12) days and that of the placebo group was 15 (95% CI 13-19) days. The rate ratio of recovery for remdesivir vs placebo was 1.32 (95% CI 1.12-1.55; *P*<.001), which demonstrated the superiority of remdesivir. In terms of the HR for death, there was no significant difference between the remdesivir and placebo groups with an HR of 0.70 (95% CI 0.47-1.04).

The remdesivir trial of Beigel et al [14] is essential to evaluate the efficacy of remdesivir, as it had a large sample size of 1059 patients under a well-designed randomized controlled trial scheme. In terms of the data analysis, Beigel et al [14] only reported the median recovery time without a *P* value. From the second figure in Beigel et al [14], the Kaplan-Meier curves of cumulative recoveries are initially intertwined and then diverge, so other percentiles of the time to recovery would provide more information on the efficacy of remdesivir. Meanwhile, a global and robust measurement, the restricted mean time to recovery (RMTR), can help to quantify the treatment efficacy in a more comprehensive way [16,22-26].

The upper panel of Table 6 presents the RMTRs up to day 30 for both the remdesivir and placebo groups. The RMTRs were 14.5 days and 17.2 days for remdesivir and placebo, respectively, indicating that patients with remdesivir on average had 2.7-day gains of recovery with 30-day follow-ups. The difference in RMTRs was statistically significant with *P*<.001, demonstrating the superiority of remdesivir. This is consistent with the original analysis in terms of the rate ratio of recovery [14]. Meanwhile in the bottom panel of Table 6, more percentiles of the time to recovery were reported with *P* values. The early difference for remdesivir vs placebo in the recovery time at the 25th percentile was –1 (95% CI –3 to 0; *P*=.65), which was not statistically significant. However, the differences manifested to be statistically significant later; for example, the 30th to 60th percentiles of the recovery time in the remdesivir group were all significantly shorter than those in the placebo group. It is reasonable for the treatment to take effect after a certain length of follow-up.

**Table 6:** The RMTR and percentiles of the time to recovery based on the reconstructed data from the second figure in Beigel et al [14].

Statistical measure		Remdesivir	Placebo	Difference (95% CI)	*P* value
RMTR^a^ (up to day 30)		14.5 (13.6-15.5)	17.2 (16.1-18.2)	–2.7 (–4.0 to –1.2)	<.001
**Percentiles of the time to recovery (95% CI)**					
	25th	5 (4-5)	6 (6-7)	–1 (–3 to 0)	.65
	30th	6 (5-6)	8 (7-9)	–2 (–4 to –1)	.002
	40th	8 (7-9)	11 (9-13)	–3 (–5 to –1)	.007
	50th (median)	11 (9-12)	15 (13-19)	–4 (–9 to –2)	.01
	60th	15 (13-19)	22 (20-27)	–7 (–12 to –3)	.004

^a^RMTR: restricted mean time to recovery.

## Discussion

When designing and conducting a clinical trial for new treatment, particularly for the COVID-19 pandemic without knowing much about the clinical outcomes, many things can go wrong if the design is not well thought out, the trial is not carefully conducted following the protocol, or the analysis is not properly carried out. Critical issues with such trials include but are not limited to the end point selection, the type I error rate control, double blinding or open label, early termination of a trial, the validity of the PH assumption in a Cox model, and assumptions for statistical tests and models. In contrast to searching for a needle in a haystack, the trial design should be more targeted, focused, and tailored for specific needs of patients with COVID-19 and particular disease characteristics and severities [32].

Given the emergency and the fast spread of the coronavirus around the world, it is crucial to design the right clinical trial and accelerate the development of a new treatment. With the high speed of enrollment and urgency of the trial outcome, it appears to be difficult to carry out any adaptation during the trial conduct. The trial outcomes unfold so fast that any adaptation may not be able to catch up with the speed of recruitment.

As a summary, our recommendations for COVID-19 trials are:

1. Adopt death as a single end point for patients hospitalized with severe COVID-19 or live discharge from the hospital for patients with moderately severe COVID-19
2. Conduct the gold standard trial scheme: a randomized, double-blind, controlled trial with equal randomization; 1:2 or 1:3 allocation ratio for control vs treatment
3. With multiple agents tested in one trial, allow the trial to drop certain treatment due to futility or toxicity
4. Adopt the RMST as the metric to quantify the treatment effect when the PH assumption is not satisfied; otherwise, standard approaches using the HRs and log-rank tests should be used
5. Control the type I error rate: Any sample size alternation during the trial must be planned and evaluated in advance with a strict control of the false-positive rate.
6. ITT analysis (or its modified version) is recommended for the final analysis.

Although adaptive design has gained much popularity and is playing an increasingly important role in clinical trials, particularly in oncology, the advantages of adaptive design may be mitigated to a large extent under such a fast patient enrollment because the impact of any adaptation may be too slow to manifest before the trial is completed. In such cases, the CONSORT (Consolidated Standards of Reporting Trials) statement [33,34] can provide a general guideline for the trial design and conduct. As a result, our recommendations follow the gold standard scheme of conventional trial design without much adaptation ingredient, which may help investigators to discriminate different treatments and identify the effective ones in an efficient way.

## Abbreviations

CONSORT: Consolidated Standards of Reporting Trials
COVID-19: coronavirus disease
HR: hazard ratio
ITT: intent-to-treat
PH: proportional hazards
RMST: restricted mean survival time
RMTI: restricted mean time to improvement
RMTR: restricted mean time to recovery
WHO: World Health Organization
